# Small bowel angiomyxoma causing intussusception: Case report and review of the literature

**DOI:** 10.1016/j.ijscr.2019.05.043

**Published:** 2019-05-31

**Authors:** Hussam M. Mousa, Suhail Al-Salam, Fikri M. Abu-Zidan

**Affiliations:** a Department of Surgery, College of Medicine and Health Sciences, UAE University, Al Ain, United Arab Emirates; b Department of Surgery, Al Ain Hospital, Al Ain, United Arab Emirates; c Department of Pathology, College of Medicine and Health Sciences, UAE University, Al-Ain, United Arab Emirates

**Keywords:** Benign, Bowel tumors, Intussusception, Myxoma

## Abstract

**Introduction:**

Angiomyxoma-related intussusception in adults is extremely rare. Herein, we report an adult man who presented with mechanical small bowel obstruction caused by ileo-colic intussusception triggered by an angiomyxoma of the terminal ileum and review the literature on this topic.

**Presentation of case:**

A-40-year-old man was referred to Al-Ain Hospital with a clinical picture of small bowel obstruction. He has no previous abdominal surgery. Clinical examination showed a distended soft abdomen, and hyperactive bowel sounds. Abdominal ultrasound and computed tomography scan showed a doughnut sign confirming the presence of ileo-colic intussusception as the cause for small bowel obstruction. At laparotomy, a mass was found in the right iliac fossa. Right hemi colectomy was performed with ileo-colic anastomosis. An ileal pedunculated mass triggered the intussusception. Histopathology of the mass was diagnostic of an angiomyxoma of small bowel. Echocardiogram showed no atrial synchronous myxoma. The patient was discharged home with good general condition. At three years follow up, the patient remained asymptomatic without evidence of recurrence.

**Conclusions:**

Myxoma of small bowel should be included in the differential diagnosis of small bowel obstruction in the young age group particularly if the diagnosis of intussusception was made preoperatively.

## Highlights

• Myxomas of the small bowel are very rare.
• They usually present with mechanical small bowel obstruction caused by an intussusception.
• They mainly occur in females in their forties.
• Surgical resection is always indicated.
• They are usually solitary and do not metastasize.

## 1 Introduction

Benign small bowel tumors are uncommon with an indolent behavior [1,2]. They usually present clinically with obstruction or bleeding [2]. Intussusception of the small bowel in adults is rare if compared with children. It is usually secondary to a small bowel pathology as a trigger point [2,3]. Myxoma of the small bowel is very rare. It is usually solitary, but multiple discrete myxomas in a single small bowel loop was previously reported [4]. Herein, we report an adult man who presented with mechanical small bowel obstruction. This was caused by an ileo-colic intussusception triggered by an angiomyxoma of the terminal ileum. This is to the best of our knowledge, is the ninth case of benign small bowel myxoma in the medical literature, and the second presented as ileocecal intussusception [5,6]. Furthermore, we have reviewed the literature on this topic. This work has been reported in line with the SCARE criteria [7].

## 2 Presentation of case

**Fig. 1:**
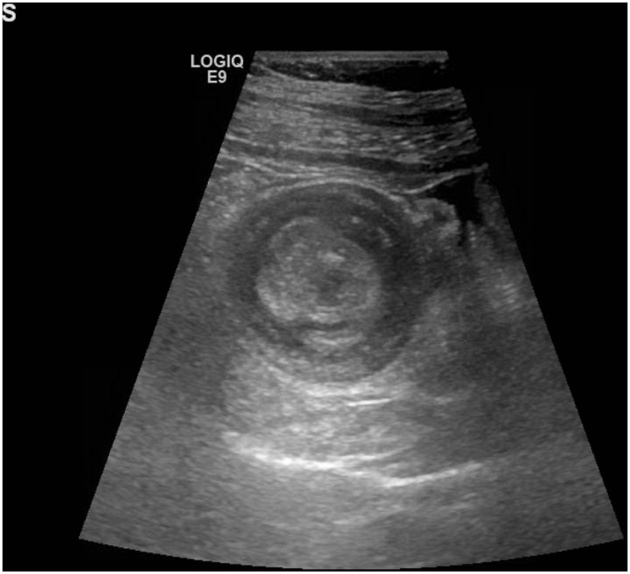
Abdominal ultrasound, transverse view, revealed doughnut or bull s’ sign, a pattern of intussusception. The patient gave his written consent to report this case and his clinical images.

**Fig. 2:**
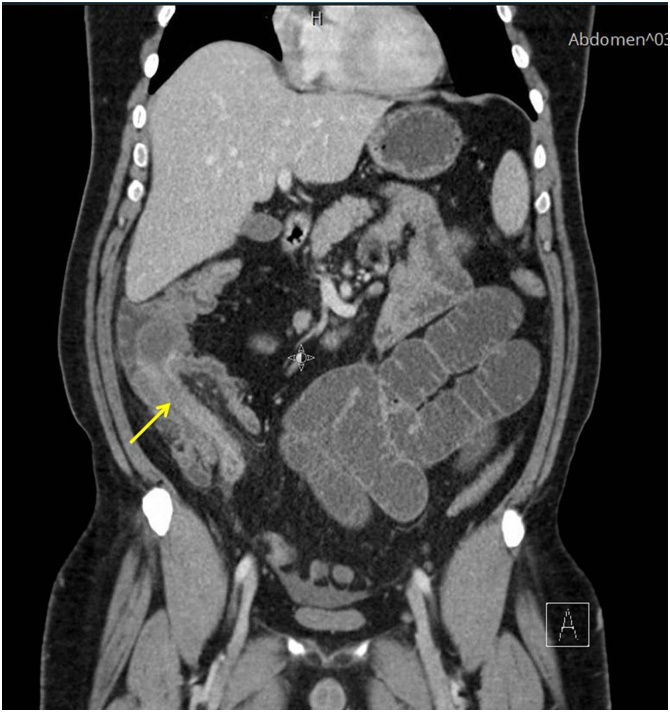
Coronal CT Scan with intravenous contrast demonstrating the intussusception of the small bowel into the ascending colon with the characteristic of double configuration of the intestinal wall (yellow arrow).

**Fig. 3:**
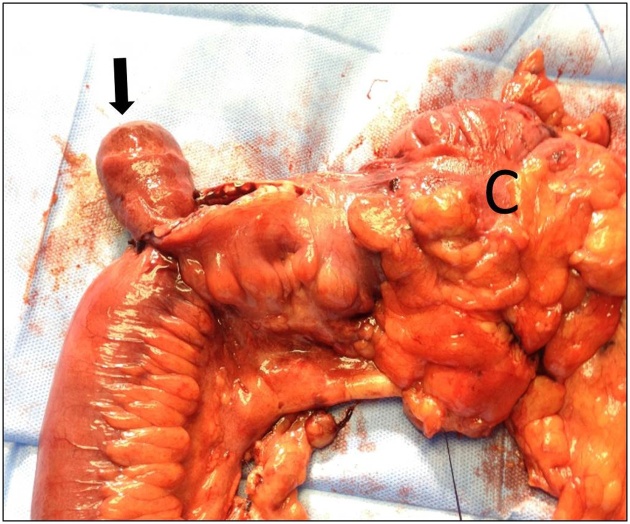
Surgeon performed right hemicolectomy for ileocolic intussusception due to polypoidal tumor in the terminal ileum measuring 59 × 35 × 30 mm (black arrow). C = caecum.

**Fig. 4:**
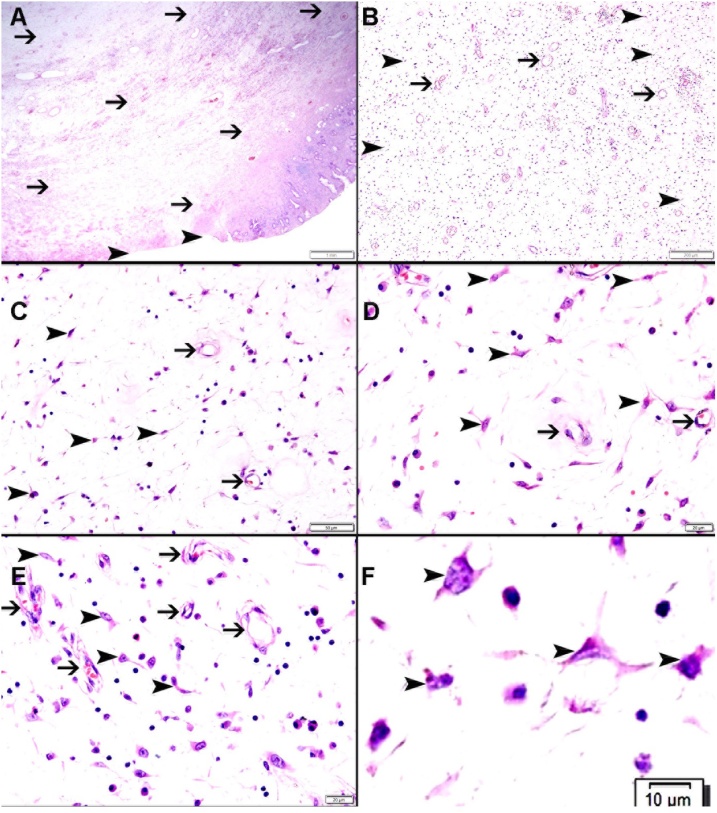
A. Representative section from the mass showing focal ulceration of colonic mucosa (arrowhead) with lightly cellular tumor involving the submucosa (thin arrow). B. Showing haphazardly arranged thin-walled capillaries and venules (thin arrow) surrounded by myxoid stroma (arrowhead). C–E. Showing thin-walled capillaries and venules (thin arrow) surrounded by myxoid stroma consisting of scattered spindle and star- shaped cells (arrowhead). F. Showing myxoid stroma with scattered star-shaped cells (arrowhead).

A 40-year-old man presented to the Emergency Department of Al-Ain Hospital complaining of generalized abdominal pain, distension, and repeated vomiting for three days. He had no previous abdominal surgery. On examination, his blood pressure was 170/80 mmHg, his pulse was 84 beats per minute, and his temperature was 37.2 °C. There was no abdominal scars or hernia defects. The abdomen was lax, grossly distended and tender. Bowel sounds were hyperactive. Digital rectal examination showed blood stained-stool. His white blood cell count was 6.2 × 10 ^9^ /L, his CRP was 6.51 mg/L. Erect abdominal X-Ray showed multiple air fluid levels in the small bowel. Abdominal ultrasound revealed distended small bowel loops and a doughnut sign in the ileo-cecal area (Fig. 1). Abdominal computed tomography scan with intravenous and oral contrast was suggestive of mechanical small bowel obstruction due to ileo-colic intussusception (Fig. 2). Laparotomy revealed a mass in the right iliac fossa with invagination of the terminal ileum into the cecum. Intra-operative reduction of the intussusception was achieved. There was intra luminal soft tissue mass in the terminal ileum 3 cm proximal to the ileocecal junction having a pedicle in the anti-mesenteric border. Right hemi-colectomy was performed with primary side-to-side ileo-colic anastomosis in 2 layers using 3/0 PDS. The excised ileo-colic segment showed a single, large polyp in the terminal ileum, which triggered the ileal invagination into the cecum (Fig. 3). Histopathology showed a sub-mucosal polypoidal tumor which was composed of myxoid stroma with proliferation of small blood vessels. There were associated lymphocytes and eosinophils. No atypia or malignancy was seen. This was consistent with a benign angiomyxoma (Fig. 4). The patient had an uneventful recovery and was discharged home on the 5^th^ post-operative day. At three years follow up, the patient remained asymptomatic without evidence of recurrence.

## 3 Discussion

Benign tumors of the small bowel are rare. Adult intussusception causes 1% of bowel obstruction, and 5% of intussusception [8,9]. Small bowel myxoma complicated by intussusception is extremely rarer [1,[4], [5], [6],[10], [11], [12], [13]] Table 1. Myxoma is a tumor of mesenchymal origin. It is composed of loose textured slimy tissue of stellate cells, reticulin fibres, and mucoid substance [5]. It is found in a variety of tissues mainly skin, soft tissue and heart [5,13]. In the intestine, myxomas usually have pedicles projecting into the lumen and triggering intussusception. These tumors do not metastasize outside their location except for cardiac myxoma. Cardiac myxomas are not true myxomas but a variant cardiac sarcomas [5,13]. Local recurrence has been reported in limited excision and close margins [5]. There was one report of synchronous intestinal and cardiac myxomas [13]. To the best of our knowledge, our patient is the ninth case of small bowel myxoma in the literature.

**Table 1:** Small bowel myxomas reported in the literature.

Author	Ref	Year	Country	Gender	Age (yrs)		Main presenting symptoms	Duration (days)	Management	Outcome	Pathology
Bourgett et al	6	1938	France	Female		20	Pain, absolute constipation	15	Segment resection SB	Recovered	fibromyxoma Ileum
Brachetto et al	10	1939	Argentina	Male		54	Acute abdominal pain	few days	Segment resection SB	Recovered	Myxoma ileum
Larimore et al	1	1941	USA	Female		48	Pain, nausea, vomiting	2	Segment resection SB	Recovered	Myxofibroma ileum
Sullivan et al	11	1942	USA	Female		44	Pain, nausea, distention	2	Segment resection SB	Recovered	Myxofibroma jejunum
Stout et al	12	1948	USA	Female		68	Pain, nausea, distention	42	Segment resection SB	Recovered	Myxoma ileum
Weinberg et al	4	1956	USA	Female		40	Pain, nausea, vomiting, no distention	2	Segment resection SB	Recovered	Myxoma ileum
Wang et al	13	2003	USA	Female		47	Pain, absolute constipation	3	Segment resection SB	Recovered	Myxoma ileum
Varsamis et al	5	2013	Greece	Female		44	Pain, nausea, distention	2	Segment resection SB	Recovered	Myxoma ileum
Mousa et al	Current case	2019	UAE	Male		38	Pain, nausea, distention, blood stained diarrhea	2	Right hemi- colectomy	Recovered	Myxoma ileum

Table 1 shows the reported cases in the literature. The female to male ratio was 7:2 (3.5). The median (range) age of the patients was 44 (22–68) years. Eight out of nine patients (88.8%) had ileal myxoma, while one (11.2%) had a jejunal myxoma. All patients presented as mechanical intestinal obstruction. Two patients [6] (22.2%) had ileocecal intussusception, both were males. All cases of ileal myxoma presented with intussusception, while the jejunal one [11] had small bowel obstruction without intussusception. All the nine cases needed surgical bowel resection with primary anastomosis, all survived and had a favorable outcome.

The diagnosis of intussusception in adults is difficult because of the vague abdominal symptoms. Complete acute bowel obstruction occurs in about 20% of the cases [8]. In our review, the pre-operative diagnosis of intussusception was reached only in three cases (33.3%) [4,5]. Abdominal X Ray was non-specific in all cases. Barium meal and follow through may show coiled-spring configuration of the intussusception of the ileum [4]. Abdominal CT scan is very accurate [5,9,13]. A combination of abdominal ultrasound and CT scan was useful in reaching the diagnosis in our case. Nevertheless, the decision for surgery is mainly clinical [14]. 90% of adult intussusception is secondary to intra luminal pathology [9,15]. They are malignant in 25% of enteric intussusception, and in 70–80% of colo-colic intussusception [3,9,14,15]. On the contrary, intussusception in children is idiopathic in more than 90% of the cases [8,9]. Unlike adults, the radiological reduction is the treatment of choice in children [9]. Radiological or endoscopic reduction for intussusception in adults is not an option; patients need surgery even if intussusception was diagnosed pre-operatively. Sarr et al recommended pre-operative reduction for enteric and ileocolic intussusception to avoid emergency surgery with its associated complications as well as performing a radical cancer surgery if needed [8]. Intra operative reduction is limited to intussusception involving long segment of small bowel to reduce the risk of extensive resection and its related complications [9]. It is recommended to avoid intra-operative reduction of colo-colic intussusception because of the high risk of malignancy and to perform instead an en-block resection [9].

## 4 Conclusions

Myxoma of small bowel is a rare disease; it should be included in the differential diagnosis of small bowel obstruction mainly in the presence of intussusception in young patients.

## Conflicts of interest

None declared by all authors.

## Funding

None.

## Ethical approval

The patient gave his written consent to report his case and publish his clinical images. According to Al-Ain Hospital Ethics Committee regulations, case reports of four and less do not need an Ethical approval from the Committee (an exempt) but needs a specific consent form that should be directly signed by the each patient approving publishing their data and clinical images. Any case series of five patients and more needs an approval of our Committee. We have obtained this written consent from our patient abiding with the regulations of our Committee. The senior and corresponding author of this manuscript is the Chair of the Human Research Ethics Committee of UAE University and confirms this fact.

## Consent

This is a case report and an exempt from a full Ethical Committee review. The patient gave his written consent to report his case and publish his clinical images. This has been explained in detail above.

## Author contributions

Hussam Mousa was the treating surgeon, had the idea, read the literature, wrote the first version and approved the final version of the paper. Suhail Al-Salam was the pathologist who reviewed the slides, prepared the pathology figure, and approved the final version of the paper. Fikri Abu-Zidan participated in the idea, edited the first version and approved the final version of the paper.

## Registration of research studies

NA.

## Guarantor

Fikri M. Abu-Zidan, Chair, Human Research Ethics Committe, UAE University, Professor; Acute Care Surgeon, Department of Surgery, College of Medicine and Health Sciences, UAE University, Al-Ain, United Arab Emirates.

## Provenance and peer review

Not commissioned, externally peer-reviewed.
